# Peptide-Enabled Nanocomposites Offer Biomimetic Reconstruction of Silver Diamine Fluoride-Treated Dental Tissues

**DOI:** 10.3390/polym14071368

**Published:** 2022-03-28

**Authors:** Sarah Kay Woolfolk, Aya Kirahm Cloyd, Qiang Ye, Kyle Boone, Paulette Spencer, Malcolm L. Snead, Candan Tamerler

**Affiliations:** 1 Bioengineering Program, University of Kansas, 1132 Learned Hall 1530 W, 15th Street, Lawrence, KS 66045-7609, USA; sarah.vanoosten@ku.edu (S.K.W.); aya.cloyd@ku.edu (A.K.C.); pspencer@ku.edu (P.S.); mlsnead@usc.edu (M.L.S.); 2 Institute for Bioengineering Research, University of Kansas, 5109 Learned Hall 1530 W, 15th Street, Lawrence, KS 66045-7609, USA; yeq@ku.edu (Q.Y.); k097b443@ku.edu (K.B.); 3 Department of Mechanical Engineering, University of Kansas, 3138 Learned Hall 1530 W, 15th Street, Lawrence, KS 66045-7609, USA; 4 Center for Craniofacial Molecular Biology, Herman Ostrow School of Dentistry of USC, The University of Southern California, 2250 Alcazar Street, Los Angeles, CA 90033-9062, USA

**Keywords:** bifunctional peptides, silver diamine fluoride (SDF), caries, silver binding peptide (AgBP), amelogenin-derived peptide (ADP), remineralization, biomimetic calcium phosphate nanocomposite interface

## Abstract

Caries is the most ubiquitous infectious disease of mankind, and early childhood caries (ECC) is the most prevalent chronic disease in children worldwide, with the resulting destruction of the teeth recognized as a global health crisis. Recent the United States Food and Drug Administration (FDA) approval for the use of silver diamine fluoride (SDF) in dentistry offers a safe, accessible, and inexpensive approach to arrest caries progression in children with ECC. However, discoloration, i.e., black staining, of demineralized or cavitated surfaces treated with SDF has limited its widespread use. Targeting SDF-treated tooth surfaces, we developed a biohybrid calcium phosphate nanocomposite interface building upon the self-assembly of synthetic biomimetic peptides. Here, an engineered bifunctional peptide composed of a silver binding peptide (AgBP) is covalently joined to an amelogenin derived peptide (ADP). The AgBP provides anchoring to the SDF-treated tooth tissue, while the ADP promotes rapid formation of a calcium phosphate isomorph nanocomposite mimicking the biomineralization function of the amelogenin protein. Our results demonstrate that the bifunctional peptide was effective in remineralizing the biomineral destroyed by caries on the SDF-treated tooth tissues. The proposed engineered peptide approach offers a biomimetic path for remineralization of the SDF-treated tissues producing a calcium phosphate nanocomposite interface competent to be restored using commonly available adhesive dental composites.

## 1. Introduction

No infectious disease is more common than dental caries. A factor in its prevalence is the maternal transmission of cariogenic microbiota [1,2]. Early childhood caries (ECC) is widely recognized as a global health crisis, with dental caries still the most prevalent chronic disease in children worldwide [3,4,5,6,7,8,9,10,11,12]. Impacting young children under age five, ECC is characterized by the presence of one or more carious lesions of the primary teeth. ECC has a higher prevalence in children of lower socioeconomic groups with limited access to dental care [9,13,14,15]. In the United States, the prevalence of ECC is estimated to be between 3 and 6% of all pre-school aged children, a value which is consistent with literature reviews confirming rates from 1–12% in the most developed countries worldwide [9,14,16,17]. However, the worst health outcomes are disproportionately skewed to certain populations as the risk of ECC in disadvantaged populations and in less developed countries can be as high as 70% [9,13,14,18]. Once a child develops carious lesions, the disease becomes more difficult and more expensive to control—rapid disease progression is common without immediate professional intervention. As the ECC disease progresses, treatment options diminish and are often very costly. Children with severe early childhood caries must commonly be treated under general anesthesia [5,18,19]. Due to the high cost and potential comorbidities, general anesthesia may not be an option for all children. More devastating to the development of the dentition and growth of the jaws is that the current standard of care in advanced ECC cases recommends premature extraction or extensive dental restorations. Early extraction of the primary teeth can alter jaw growth, leading to the failure of the remaining and adult replacement teeth to work together effectively during chewing. This can lead to consequential changes in micro- and macro-nutrition that adversely impact child health across a lifetime [18,20]. Therefore, alternative treatment options that circumvent the cascade of failure described above have been the focus of attention in the dental public health community.

Fluorides have proven useful to slow the progression of dental caries by replacing the hydroxyl group with fluoride in the hydroxyapatite biomineral of the teeth, thereby inhibiting the carious demineralization of teeth [21,22,23]. Recently, silver diamine fluoride has gained attention as a safe, accessible, and inexpensive approach to arrest caries progression in children with ECC [3,4,8,24,25,26]. SDF has been used worldwide for decades but was approved for dental use by the FDA in 2014 [27,28]. Although the exact mechanism of action has not been resolved, silver ions deposited on the dental tissues have notable antimicrobial properties [29,30]. Recently, silver microwires have been described in teeth treated with SDF―presumably the microwires are replacing defects that are a result of caries-provoked demineralization [31].

Unlike surgical interventions which require skilled professionals, SDF treatments can be applied by a wider range of health care providers [32]. SDF works by limiting caries progression and protecting teeth from further degradation. As of 2016, the 38% SDF solution was granted breakthrough therapy designation by the U.S. Food and Drug Administration for use in treating ECC [26,28,33,34]. Numerous case studies have shown the overwhelming benefit of single or bi-annual SDF treatments for caries arrest, with a focus for use of SDF in the primary teeth of children affected by ECC [3,8,26,28,33,35,36]. SDF is inexpensive, the application takes minutes, and it does not require significant patient cooperation. Only rare minor gingival irritation and no serious adverse events are associated with the use of SDF [37].

A side effect of SDF is black staining of the treated demineralized or cavitated surfaces due to the deposition of silver metal and ions [3,8,28,35,36]. This side effect has limited the widespread adoption of SDF treatment for ECC and caries arrest. The loss of aesthetics was documented by Crystal et al., who interviewed parents of children qualified to receive SDF treatment and found that roughly one third of parents found the treatment unacceptable under any circumstances [38].

SDF is recommended for use on carious lesions in primary teeth and root caries and may prevent the progression of the infection [39]. In addition, SDF treatment is recognized not to reduce immediate bonding performance of resin composite to non-carious materials however further studies are recommended to investigate the effect of SDF on dentin collagen at the dentin-adhesive interface [40].

Recent advances in peptide design have resulted in predictable-, tunable-bifunctional peptides. These peptides can self-assemble on complex surfaces, i.e., surfaces ranging from metal to minerals, to produce an biomimetic interface serving to modulate desired functions [41,42,43]. Our group, and others, have identified peptides using combinatorial biology, such as phage display technology, and enhanced their properties by computational methods, such as bioinformatics, to specifically target solid materials including metals, metal oxide, minerals, and polymers [42,43,44]. We demonstrate that different peptides can be deployed to combat bacteria, direct remineralization of defective enamel and dentin, and as key bioactive components of novel dental biomaterials and imaging probes, facilitate tissue integration [44,45,46,47,48,49,50,51,52]. We previously identified a silver binding peptide (AgBP; EQLGVRKELRGV) and demonstrated its self-assembly properties on silver nanoparticles and silver surfaces in the presence of covalently coupled additional bioactive moieties for bioimaging and biosensing applications [48,53,54].

Natural proteins demonstrate that polypeptide chains can be integral to hard tissue mineralization and remineralization. Amelogenin, the most abundant enamel matrix protein, is recognized to exert a dominant role during enamel biomineralization by hydroxyapatite mineral [55,56,57,58]. Using phage display technology, our group identified hydroxyapatite-binding peptides (HABPs) and demonstrated their ability to control both the kinetics and crystallite morphology of calcium phosphate deposition as hydroxyapatite. [59]. To understand which amelogenin domain(s) likely participates in biomineralization, a knowledge-based bioinformatic design was applied. We created HABP scoring matrices and an algorithm to identify regions of similarity between natural proteins and biocombinatorially selected peptides [60]. The knowledge acquired from the biocombinatorially selected peptides integrated with detailed experimental peptide characterization was used to identify the potential functional domains in the amelogenin protein which could be used for tissue repair and restoration [44]. Therefore, in identifying these functional domains within a naturally occurring protein such as amelogenin, our approach includes these functional domains in a biomimetic peptide sequence to direct biomineralization at the carious region. Our bioinformatics search identified several amelogenin domains within the full-length amelogenin protein predicted to participate in controlling biomineralization, and these were referred to as amelogenin-derived peptides (ADPs) [60,61]. ADP5 demonstrated capacity to favorably modulate both the kinetics and crystallite morphology of hydroxyapatite formation, and ADP5 achieved the modulation in a manner that closely resembled the kinetics function of the full length amelogenin protein [44]. Therefore, we select a version of ADP5 as part of our chimeric peptide design described herein [44].

In this study, using an engineered peptide, we explore specifically driving nanocomposite formation for biomimetic reconstruction of SDF-treated tooth tissues in order to mitigate the negative side effect, i.e., black staining, associated with SDF treatment. Starting with a 22-amino acid long human amelogenin-derived peptide shADP, it was engineered further to allow for improved water solubility, while still preserving the peptide’s function for rapid and robust mineral forming properties [44,61]. The resulting shorter sequence, denoted as shADP5 (SYEKSHSQAINTDRT), was selected for the current studies due to its increased solubility, greater ease of synthesis, and reduced cost. Having selected shADP5 and AgBP for remineralization and silver binding, respectively, we next engineered novel bifunctional peptide with computational analysis of selected spacer sequences that combine the functions of binding to SDF-treated dental tissue and directs remineralization at that interface. We demonstrate that the engineered bifunctional peptide performs both of these roles. Our work highlights the potential therapeutic role that bifunctional peptide can offer by allowing the peptide to bind to the silver deposits while also rebuilding damaged dental tissues, adding a new layer of protective calcium phosphate biomimetic mineral. Nanocomposites, often with calcium phosphate nanoparticles, have been studied for inhibiting caries [62]. This peptide-based biomimetic nanocomposite approach adds a new capacity to the recent FDA approved use of silver diamine fluoride (SDF) treatment of arresting caries progression with the addition of peptide-mediated remineralization directed through interactions with silver-treated surfaces. Furthermore, our approach mitigates SDF’s adverse effect, i.e., black staining of the treated carious lesion.

## 2. Materials and Methods

### 2.1. Materials

*N,N*-Dimethylformamide (DMF, 99.8%), diethyl ether (99%), trifluoroacetic acid (99%), triisopropylsilane (98%), and sodium azide (99%) were obtained from Sigma-Aldrich (St. Louis, MO, USA). *N*-methyl morpholine (NMM), Rink amide resin, Fmoc-resin, Fmoc-amino acid building blocks, and 2-(1H-benzotriazole-1-yl)-1,1,3,3-tetramethyluranium hexafluorophosphate (HBTU) were purchased from AAPPTec LLC (Louisville, KY, USA). 1,2-ethanedithiol (95%) and hydroxymethyl (Tris) aminomethane hydrochloride (99%+, extra pure) were purchased from Acros Organics (Carlsbad, CA, USA). Phenol (89%) was certified and received from Fisher Scientific (Fair Lawn, NJ, USA). Calcium chloride dihydrate (99.7%), potassium phosphate monobasic (99.9%), and potassium chloride (99.7%) were certified ACS grade and obtained from Fisher Scientific (Fair Lawn, NJ, USA). Hydrogen peroxide (30%), %sulfuric acid (96.5%), ammonium fluoride (99.3%), sodium phosphate dibasic anhydrous (99.8%), and sodium chloride (99.9%) were certified ACS grade and purchased from Fisher Chemical Industries (Carlsbad, CA, USA). AgNO_3_ was purchased from Consolidated Chemical and Solvents LLC (Quakertown, PA, USA). A 38% SDF solution Dengen Caries Arrest was obtained from Dengen Dental (Bahadurgarh, Haryana, India). All chemicals were used without further purification. We received 1 ku FastAP thermosensitive alkaline phosphatase from Thermo Fisher Scientific (Waltham, MA, USA), where one unit (u) is the amount of enzyme required to dephosphorylate the 5′termini of 1 μg of linearized pUC57 DNA in 10 min at 37 °C in FastAP buffer, per its certificate of analysis. Single-side polished boron-doped Si (111) wafers (resistivity 3.0–6.0 Ω·cm) were purchased from Virginia Semiconductor (Fredericksburg, VA, USA).

### 2.2. Peptide Design

The initial selection of the silver-binding peptide sequence using FliTrx (Invitrogen, Fair Lawn, NJ, USA) bacterial surface display was previously reported by our group [53]. The AgBP2 (EQLGVRKELRGV) 12 amino acid sequence was selected for use in bifunctional peptide design [48,53,54]. Binding confirmation studies were completed using DsRed-AgBP2, as used in previous studies, to visualize binding to the SDF-treated tooth samples by the fluorescent reporter, DsRed protein. Production of DsRed-labeled solid-binding peptides was reported in previous publications [63,64]. For the bioactive remineralization portion of this work, we selected a peptide derived from human amelogenin protein, shADP5 (SYEKSHSQAINTDRT) [44,61].

The studies herein designed a novel bifunctional peptide, shADP5-AgBP2, by combining two individually identified and characterized peptides. The two peptide sequences were designed to join by a short, flexible spacer designed using biocomputational modeling integrated with experimental studies to ensure individual activities were maintained in the bi-functional peptide. The pairwise displacement of alpha carbons of the respective peptide domains were minimized among a selection of candidate spacer sequences. PEP-FOLD3 was used to generate candidate chimeric peptide structures [65]. The structures were compared pairwise for each structure between groups for the sum of squares of distances between corresponding alpha carbons, calculated by RRDistMaps [66]. These comparisons were normalized by the internal mean distance for the domain-only structures. The mean distance difference is expressed as the percentage increase over the self-group mean distances of the individual domains. The sum of this difference was minimized to select a bifunctional peptide sequence, representing the smallest change in computationally predicted average alpha carbon distance.

### 2.3. Peptide Synthesis

The shADP5-AgBP2 peptide was synthesized using standard Fmoc solid-phase peptide synthesis on the AAPPTec Focus XC (AAPPTec, Louisville, KY, USA) automated peptide synthesizer, following previous published protocols [50,51,67]. Following synthesis, the peptide was cleaved from the resin and side-chain-deprotected using a cleavage cocktail (trifluoroacetic acid/phenol/ethanedithiol/triisopropylsilane/DI water (87.5:5:2.5:2.5:2.5) for 2 h and precipitated in cold ether. Crude peptide purification was performed by semi-preparative reverse-phase high-phase liquid chromatography (HPLC) on a Waters system, composed of a Waters 600 controller and a Waters 2487 Dual Absorbance Detector, using a 10 μm C-18 silica Luna column (250 mm × 10 mm, Phenomenex Inc., CA, USA). The mobile phase was 94.5% HPLC-grade water, 5% chromplete acetonitrile, 0.1% trifluoroacetic acid (phase A), 99.9% chromplete acetonitrile, and 0.1% trifluoroacetic acid (phase B). Lyophilized peptide was dissolved in 3 mL of phase A, then purification was executed in a 0.5% phase B·min^−1^ linear gradient from 5% B to 85% B, with a 3 mL·min^−1^ flow rate at room temperature and detection at 254 nm. Fractions collected were verified via analytical HPLC on a Shimadzu system, composed of a Shimadzu LC-2010 HT liquid chromatograph and an SPD-M20A prominence diode array detector, using a 5μm C-18 silica Luna column (250 mm × 4.6 mm, Phenomenex Inc., Torrance, CA, USA). The mobile phase was 99.9% HPLC-grade water and 0.1% trifluoroacetic acid (phase A), and 100% acetonitrile (phase B). The system operated on a linear gradient under these conditions: 1 mL min^−1^ flow rate; detection at 254 nm; 10 μL sampling loop; 40 °C temperature. The purified bifunctional shADP5-AgBP2 peptides were then lyophilized and stored at −20 °C.

### 2.4. Peptide Functionalization of Dental Tissue

Lyophilized peptide stocks were selected and reconstituted in Milli-Q (resistance, >18 MΩ) water immediately prior to experiments with both silver-coated silica substrates and slabs of dental tissue. Stock solutions were prepared to ensure a final maximum concentration of 100 μM peptide could be achieved. Silica substrates or slabs of dental tissue were submerged into a solution containing 50 μM peptide and allowed 2–4 h gentle shaking at room temperature to ensure the binding of AgBP. This excess concentration was used to ensure ample peptide available.

### 2.5. Silver-Coated Silica Substrates

To confirm the selectivity and specificity for silver binding of bifunctional peptides, preliminary studies were conducted on silver-coated silica substrates. Silica wafers were cut into 1 cm square pieces using a diamond scribe, and a published protocol for electrolysis deposition of silver onto silica was followed [68,69]. Briefly, the Si (111) wafers were cleansed in hydrogen peroxide and sulfuric acid solution (3:7) for 20 min, rinsed thoroughly with Milli-Q water, and dried with N_2_ gas prior to use. An etching step was employed prior to Ag deposition, where a drop of 5% NH_4_F solution was placed on the surface of the cleaned silica substrate for 30 s and removed carefully with N_2_ gas. A plating solution was prepared with 0.020 M NH_4_F and 0.010 M AgNO_3_ and placed onto the pre-etched silica substrates for about 60 s to allow for silver deposition. After the reaction occurred, the substrates were washed with Milli-Q water.

### 2.6. Preparation of Slabs of Dental Tissues

Specimens of dental tissues were prepared using a protocol modified slightly from our previous publications [45]. The specimens consisted of extracted human molars. These teeth would otherwise be discarded, no patient identifiers are associated with the teeth and thus, this is not considered human subject research. The extracted teeth were stored at 4 °C in 0.9% wt/vol NaCl containing 0.002% sodium azide. Teeth exhibiting carious lesions were selected and the root structure was removed by sectioning perpendicular to the long axis of the molar using a water-cooled low-speed diamond-blade saw (Buehler, Lake Bluff, IL, USA). The tooth was sectioned parallel to the long axis to provide 1.4 mm thick slabs. The prepared sections were stored in a modified PBS buffer (20 mM Na_2_HPO_4_, 137 mM NaCl, 2.7 mM KCl, and 1.8 mM KH_2_PO_4_, pH 7.4) at 4 °C until experimental use.

### 2.7. SDF Treatment Protocol

SDF was applied to dental tissue following the detailed guidelines published by the UCSF Caries Arrest Committee [25,34]. Briefly, the prepared slabs of dental tissue were removed from the modified PBS buffer, rinsed with Milli-Q purified water, and gently dried with compressed air to reveal suitable carious regions of the dental tissues. A single drop of commercially available 38% SDF solution was placed into a fresh plastic dish. A microbrush applicator head was dipped into the solution and touched to the side of the dish to remove excess fluid by surface tension. The SDF was applied to the dental tissue, allowed to absorb for 1 min and the treated sample was gently rinsed with Milli-Q purified water. The tissue was placed back into modified PBS buffer and incubated at 37 °C for 6 h to mimic the oral environment and to allow for SDF activity. The progressive darkening of the treated regions due to precipitation of silver and fluoride from the SDF on the dental tissue was noted.

### 2.8. Enzyme-Mediated Biomineralization

To investigate calcium phosphate nucleation facilitated by the shADP5 peptide, an alkaline phosphatase (AP)-based mineralization model was followed [44]. This model mimics the biological process wherein inorganic phosphate is cleaved from organic phosphate by naturally occurring AP and subsequently reacts with free calcium. Briefly, control samples without peptide and test samples functionalized with shADP5 were submerged in a remineralization buffer containing 14.4 mM β-glycerophosphate (β-GP) and 24 mM Ca^2+^ in 25 mM Tris-HCl at pH 7.4. Mineralization reactions were initiated by adding FastAP (thermosensitive alkaline phosphatase) to the solutions to achieve a final concentration of 1.4 × 10^−6^ g/mL and allowed to react at 37 °C for 2 h to form a calcium phosphate mineral layer on the sample surface. Samples were removed from the solution and allowed to air dry prior to microscopic and spectroscopic analyses.

### 2.9. Fluorescence and Optical Microscopy

Optical microscopic images of dental tissue and Ag-coated silica substrates were taken at 10× magnification on a Nikon SMZ800 StereoMicroscope (Nikon Instruments Inc., Melville, NY, USA) system equipped with a Q-Imaging MicroPublisher 5.0 RTV camera (Teledyne QImaging, Teledyne Photometrics, Tucson, AZ, USA). Images were processed with the Q-Capture software (Teledyne Photometrics, Tucson, AZ, USA).

Fluorescence images were acquired using a Leica TCS SPE Laser Scanning Confocal DM6-Q upright microscope (Leica Microsystems, Wetzlar, Germany) equipped with a 10× Leica Infinity corrected objective. DsRed fluorescence was captured using a laser excitation of 561 nm. Images were processed using the Leica LAS-X imaging software.

### 2.10. Mineral Characterization

To image the mineral morphology, surface coverage, and to determine the Ca/P ratios of the precipitated material, a Hitachi SU8230 Field Emission Scanning Electron Microscope (Hitachi High-Tech America, Schaumburg, IL, USA) equipped with a silicon drift EDS detector (X-Max, Oxford Instruments, Concord, MA, USA) was used. SEM imaging was completed at 5 kV and EDS measurements made with an acceleration voltage of 10 kV to improve signal. Prior to SEM/EDS analysis, samples were sputter coated with 5 nm iridium using a Quorum sputter coating system (Q150, Quorum, Laughton, East Sussex, UK). EDS analysis was performed using AZtec software (Oxford Instruments, Concord, MA, USA) and values averaged across a minimum of three regions for each sample.

## 3. Results

### 3.1. Silver Binding Peptide Assembles onto SDF-Treated Dental Tissues

Restoring diseased dental tissue requires addressing multiple challenges simultaneously. We have previously shown the specific and selective binding attributes of a silver-binding peptide (AgBP; EQLGVRKELRGV) to anchor to silver nanoparticles and surfaces [48,53,54]. The silver-binding peptide was investigated for its specific targeting and binding properties onto the SDF-treated carious dental tissues.

In our prior art, we engineered fluorescence fusion proteins with a peptide tag that self-assemble on metallic nanomaterials as bimodal imaging nanoprobes [53,54,63,64]. Engineered fusion green- or red-fluorescent proteins (GFP and DsRed, respectively) were developed by incorporating silver-binding (AgBP) and gold-binding peptides (AuBP). Fluorescent labelling using small fluorophores commonly requires additional labeling steps, whereas the fluorescence proteins with the metal-binding peptide tags as a fusion partner offer to identify the location of the peptide in a single step to monitor surface modification and the self-assembly process.

**Figure 1:**
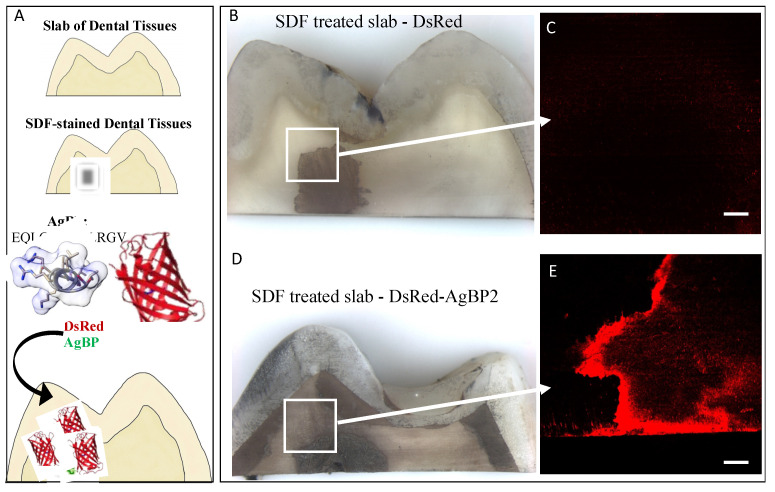
DsRed–AgBP assembled selectively on silver stains on slabs of dental tissues treated with SDF and visualized by fluorescence signal (**A**). Control, DsRed protein alone (**B**,**C**) or DsRed–AgBP2 (**D**,**E**) functionalized SDF-treated slabs were surveyed by fluorescence microscopy. Scale bars are 1000 mm for bright field images and 100 μm for fluorescence images.

Here, we employed DsRed–AgBP fusion protein for direct visualization of silver binding ability of AgBP via the DsRed fluorescence reporter protein onto the SDF-treated slabs of dental tissue shown in Figure 1. The DsRed protein without an AgBP tag was used as the control (Figure 1B,C) on the SDF-treated dental tissue and this resulted in only minor fluorescent, whereas a robust red-fluorescent signal was observed on the SDF-treated dental tissue functionalized by DsRed–AgBP (Figure 1D,E). The result confirms that the selective silver-binding property of AgBP was preserved in the presence of the fluorescent signal showing colocalizing only to the silver-treated areas following silver diamine treatment of the dental tissue. Further studies supported the interactions of the silver-binding peptide alone with the SDF using ATR-FTIR spectroscopy (Figure S1). The observed binding specificity result agrees with previous work showing AgBP in a maltose-binding-protein construct had an equilibrium dissociation constant about two orders of magnitude lower than the construct without a solid-binding peptide and about one order of magnitude for the construct with a weaker solid-binding peptide [70]. The selectivity of binding mechanisms for AgBP and other peptides have been explored outside of our group in a comparative study of solid-binding peptides on silver and gold surfaces [71]. The comparative study found that binding for peptides on the silver surface was shown to be more entropy driven, while the binding of peptides on gold surface was shown to be more driven by enthalpy.

### 3.2. Bifunctional Peptide Design Targeting SDF-Treated Dental Tissues

Building upon the observed AgBP binding to silver deposits of the SDF-treated dental tissue, we next turned to engineering a bifunctional peptide containing silver binding with a peptide driving mineral formation. For this mineral formation function, we selected an amelogenin-derived peptide, shADP5 (SYEKSHSQAINTDRT), which imparts fast remineralization kinetics as well as control of crystallite morphology in a manner similar to the full length amelogenin protein [44,61]. The ADP5 peptide had previously been shown to regulate formation of a robust hydroxyapatite mineral layer on demineralized human dentin root surfaces in a cell-free assay [44]. When combining different peptide functionality, i.e., mineralization and metal binding, the challenge to address lies in the design of a spacer between the two peptide functions that minimizes potential interference between the domains. Therefore, we next investigated several spacers of various amino acid sequence as the linking element between the shADP5 and AgBP domains. Spacers are known to influence domain activity through length and flexibility [72]. In previous studies, bifunctional peptides design which included flexible spacers (e.g., GGG, GSGGG) resulted in maintaining successful function for each peptide domain [73,74].

#### 3.2.1. Computational Analysis of Peptide Design

We use an “in-solution” structure prediction [65] to estimate the changed folding dynamics of the bifunctional peptide context compared with the single-domain peptide [66]. The structural variations examined include changes to diffusion rates between the bifunctional domain attributes and the changes to backbone conformation for each domain of the bifunctional peptide depending on the relative orientations of the domains. Since the diffusion rates for longer peptides generally decrease, bifunctional peptides diffuse slower compared with single-domain counterparts. While slower diffusion of bifunctional peptides may limit some applications, for systems in which the material surface–peptide interaction offers clear competitive advantages when surface assembly occurs, the slower diffusion becomes irrelevant. This limitation can be overcome for systems in which the material surface–peptide interaction offers competitive advantage for surface assembly. Moreover, the change in the backbone conformation may influence the free energy of adsorption and desorption when comparing single-domain and bifunctional peptides.

We searched for spacers that induce minimal change in the folding dynamics for each of the domains [75,76,77]. Our objective for spacer design is to minimize the backbone changes in computationally folded structures. We compare chimeric structures with single-domain structures for selecting the spacer sequence which minimizes the structural change.

(1) $$minimize:\;\sum^{\;}(\min\sum_{single,\;chimeric}^{Paired\;Structures\;between\;Clusters}{\left|C_{\alpha ,\;single}\left(x,\;y,z\right)-C_{\alpha ,\;chimeric}\left(x,\;y,z\right)\right|}^{2})$$

**Figure 2:**
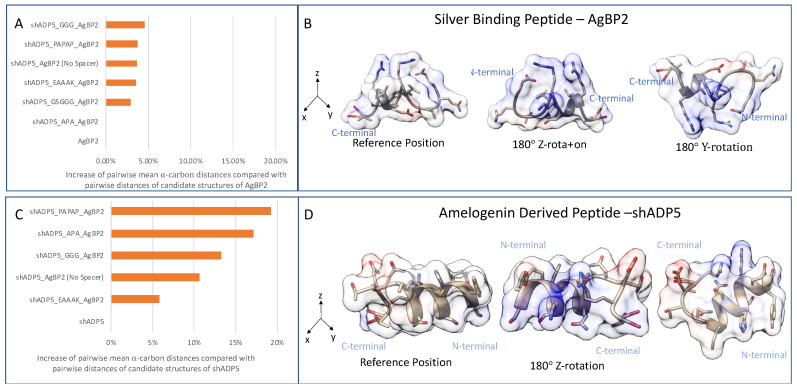
Structure similarity of bifunctional peptide domains compared with each domain in isolation for the silver-binding peptide and amelogenin-derived peptide. Panel (**A**), pairwise distance of domain structures compared with non-chimeric domain; Panel (**B**), single-domain structure views of AgBP2; Panel (**C**), similarity comparison for amelogenin-derived peptide (shADP5) chimeric; Panel (**D**), pairwise distance compared with non-chimeric domain single-domain structure views of shADP5. A 1% increase in pairwise distance would be the same as 101% increase in the mean pairwise α-carbon distance for the non-chimeric domain.

The backbone alpha-carbons compared between structures are limited to the overlapping sequence parts between the chimeric sequence and the single domain. Larger distances between alpha-carbons indicate larger folding changes for the domain, with the implication that the probability of domain function change is proportional to the folding change. We hypothesized that these distances are spacer dependent. Figure 2 shows that selected spacers result in different proximities and different orientations between the domains. The proximities and orientation between the domains strongly influence the observed peptide backbone shape changes for each individual domain. Figure 2A shows that the AgBP2 had a smaller change in backbone folding than the sh-ADP5 domain (Figure 2C). Finding which backbone folding changes are the most related to domain function changes can be addressed through systematic chimeric peptide function observations in future comparative studies.

**Figure 3:**
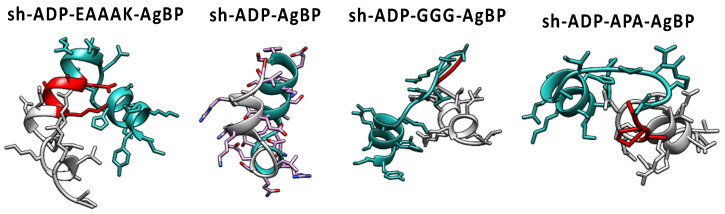
Folding pattern of the bifunctional peptide structures composed of AgBP2 and shADP5 peptide domains linked with different spacer sequences. Estimated magnitude of the folding change is provided in Table 1.

The distances between the single domain (2 cluster representatives for AgBP2 and 5 cluster representatives for shADP5) compared with the bifunctional peptides (5 cluster representatives for each sequence) is characterized by the mean sum of squares of distances between corresponding alpha carbons by cluster, as shown in Figure 2. These mean sums of squares were scaled by the single-domain comparison. The self-comparison is the mean of the pairwise distances between different cluster representatives. When these percentages were summed for both single domains for each chimeric sequence, spacer EAAAK resulted in the smallest predicted backbone change (9.4%), followed by APA (17%) and the GGG spacer (18%). Using no spacer in the chimeric sequence resulted in a summed percentage of 14%. The alpha-helical spacer of EAAAK results in the minimal predicted change in backbone conformation among spacers evaluated. Table 1 gives the summed percentages for all candidate chimeric peptide sequences. We also provide 3D models showing computationally folded structures for the four chimeric peptides with the least changes in their backbone in Figure 3.

#### 3.2.2. Functional Activity of sh-ADP5-AgBP2 on Ag-Coated Silica Substrates

Derived from the amelogenin protein, the most abundant protein associated with enamel formation in mammalian teeth, the shADP5 peptide, was selected for use in these studies based on its observed rapid kinetics for calcium phosphate precipitation as reported in previous studies [44,78]. Since the bifunctional peptide, shADP5-AgBP2, is a novel peptide, the preservation of activity contributed from each functional domain was first investigated on Ag-coated silica.

**Figure 4:**
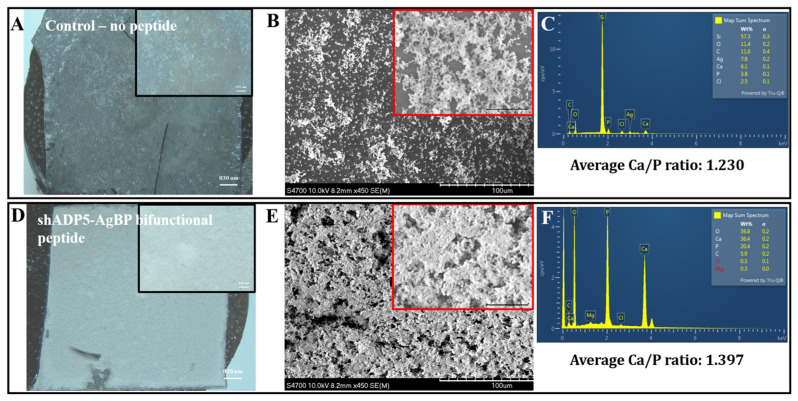
Mineral deposition on silver-coated surfaces. Ag-coated surfaces with no peptide (control, panels **A**−**C**) or 50 mM shADP5-AgBP-treated surfaces (panels **D**−**F**). Optical microscope images (**A**,**D**), SEM images (**B**,**E**), EDS spectra (**C**,**F**), and average Ca/P ratios (insert to **C**,**F**) for mineralized regions after 2 h alkaline phosphate-induced mineralization. Scale bars are 830 mm and 195 mm for insets in (**A**,**D**), or 10.0 μm and 25 μm for insets in (**B**,**E**).

Figure 4 shows representative optical images of the Ag-coated substrates with no peptide (control) or with 50 μM shADP5-AgBP. The samples were subjected to an enzyme-mediated mineralization protocol, mimicking the biomineralization processes from nature. The samples were gently washed by water and the formed mineral layer was dried overnight prior to SEM/EDS analysis. Alkaline phosphatase-based mineralization resulted in significantly higher mineral coverage in the presence of shADP5-AgBP bifunctional peptide (Figure 4D,E) compared with control samples (Figure 4A,B). EDS results confirm the formation of a calcium phosphate mineral isomorph from which we calculated the calcium/phosphate ratios to compare potential mineral compositional differences among samples. The mineral layer formed in the presence of the shADP5-AgBP bifunctional peptide had an average Ca/P ratio of about 1.40 (Figure 3F) as compared with 1.23 in control samples (Figure 3E). A wide range of distinct calcium phosphate phases exist in mineralized tissues and these phases are commonly classified by the Ca/P molar ratio [59,79,80,81]. The molar ratio of 1.40 falls between that of octacalcium phosphate and amorphous calcium phosphate with Ca/P ratios of 1.33 and 1.50, respectively. The results confirm that the bifunctional peptide, shADP5-AgBP, preserved its mineralization activity. Peptide function is highly dependent upon structure and conformation, with the spacer sequence between the two active domains playing a critical role in allowing each peptide to function to its fullest [74]. Changes to the spacer sequence may allow further optimization of the function of the shADP5 peptide or another mineral-mediating peptide to achieve a specific CaP mineral composition and morphology.

### 3.3. Bifunctional Peptide-Enabled Mineralization on SDF-Treated Dental Tissues

**Figure 5:**
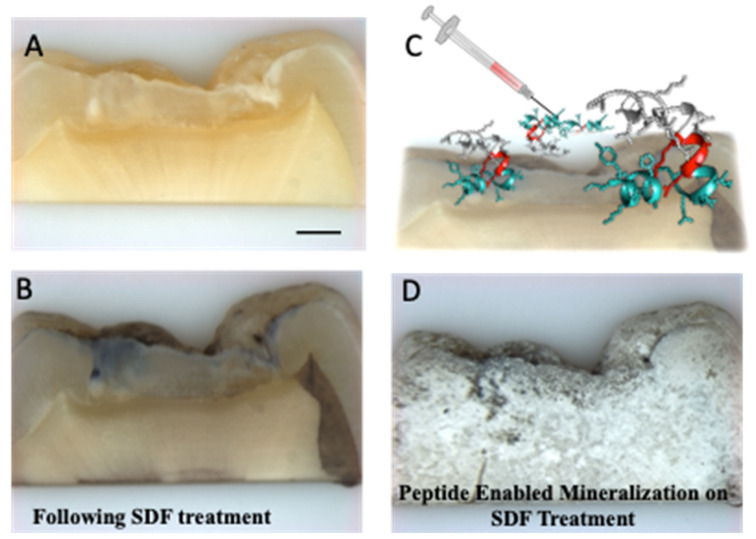
Mineralization by the shADP5-AgBP functionalized SDF-treated dental tissue. Optical microscopy images of untreated dental tissue (**A**) and SDF-treated dental tissue (**B**). A schematic of the bifunctional peptide applied to SDF-treated dental tissue (**C**) follows the colors assigned to the carbon backbone of the peptide shown in Figure 3. Mineralization enabled by the bifunctional peptide functionalized SDF treated dental tissue (**D**). Scale bar, 1000 μm.

Building upon the success of the bifunctional peptide mineralization on silver-coated surfaces, we prepared four SDF-treated slabs of carious dental tissue. Figure 5 reveals the capacity for the shADP-AgBP peptide to direct mineralization onto the SDF-treated dental tissues which was investigated for mineral coverage onto the silver-deposit-treated areas of dental tissues using bright field optical microscopy.

**Figure 6:**
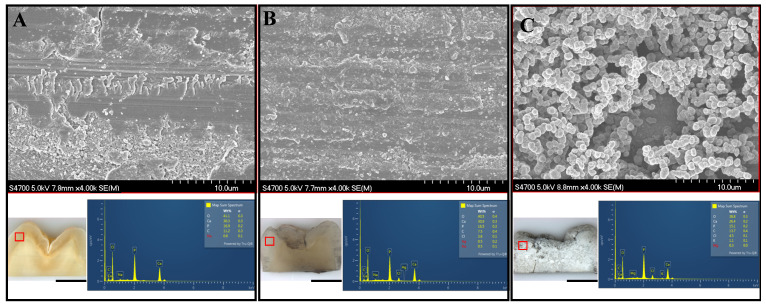
SEM images and corresponding EDS spectra from the enamel tissue of the dental slabs shown in Figure 5. Representative images are shown for: untreated sample (**A**); SDF-treated slab (**B**); shADP-AgBP peptide applied to SDF-treated silver-stained dental tissue slab (**C**). Scale bar for 10× survey images (**A**−**C**) of dental tissue slabs is 1000 μm. Representative EDS spectra to determine Ca/P averages calculated across a minimum of three unique areas for each region studied.

Treated dental tissues were analyzed using SEM for imaging and EDS for compositional analysis. Average Ca/P molar ratios were calculated for each slab of dental tissue from both SDF-treated and untreated regions of enamel and dentin, corresponding to SDF treatment of carious dental tissues, to assess mineral formation and compositional changes on the tooth samples in the presence and absence of the shADP5-AgBP2 bifunctional peptide. SEM images with EDS spectra from representative regions of each treated dental tissue are shown in Figure 6. Differences noted in the mineral morphology formed on the peptide mediated mineralized sample shown in Figure 6C were attributed to the presence of the mineral-mediating peptide shADP5. Average Ca/P ratios across treatment groups from both SDF-treated and untreated enamel and dentin regions are reported in Table 2.

The average Ca/P ratios presented in Table 2 allow for direct comparisons between two sets of sample pairs: (1) untreated control and SDF-treated control, and (2) mineralized dental tissue slab with and without shADP5-AgBP2 peptide. The formed mineral layer on dental tissue slabs leads to differences in the CaP composition across the two groups, as the first group of data is directly formed on the dental tissue, rather than forming a new mineral layer. Noteworthy is the finding that the SDF treatment resulted in slightly greater Ca/P ratios from untreated regions of the tooth compared with its untreated counterpart, with a more notable increase in the regions from the enamel tissue. However, in the treated regions of the SDF-treated control dental tissue, a significantly greater Ca/P ratio was demonstrated for both SDF-treated enamel and dentin regions, as compared with untreated regions of the same sample and from the untreated control samples. A 2021 study investigated the effects of SDF on carious primary teeth regions and found SDF treatment promoted pathologic mineral by altering physiochemical properties of the tooth structures [82]. Their detailed results include structural and elemental analysis of SDF-treated teeth which reported EDS results consistent with our findings [82]. The increased Ca/P ratio in the SDF-treated control sample indicates that in these carious lesions of the tooth, the SDF treatment creates a more stable CaP material closer resembling a calcium-deficient apatite [44,61,83].

For the enzyme-mediated remineralization group, SDF-treated dental tissues were functionalized with either 0 μM or 50 μM shADP5-AgBP2 prior to the mineralization protocol. Similar to what was observed with the Ag-coated samples (Figure 2), a significant increase in Ca/P ratio was observed in the sample functionalized with the peptide. On average, the Ca/P ratios from the mineralized layer observed in both the peptide-and SDF-treated tissue as well as the SDF-only treated tissues, were about 0.1–0.15 less than what was obtained for the untreated and peptide-functionalized Ag-coated silica samples. The presence of calcium phosphate rich minerals in the slabs of dental tissue may alter the availability of free calcium and phosphate in the enzyme-mediated mineralization reaction. However, this same trend was noted in the presence of our shADP5 peptide where the Ca/P ratio was significantly greater than samples not functionalized with peptide, a finding confirming biomineralization activity of the peptide. Differences in the mineralization may also be attributed to the anchoring effect of the AgBP2 peptide being bound to the SDF-treated regions of the dental tissue.

## 4. Discussion

With recent advances in peptide engineering that incorporate a tunable design to adapt functions, peptides offer a promising approach to favorably modulate even the most complex surfaces and interfaces. By combining shADP5 with AgBP, we engineered a novel bifunctional peptide that binds to SDF-treated, silver-containing regions on specimens of dental tissue and facilitated remineralization on the peptide-functionalized dentin and enamel surfaces. Our preliminary results highlight just one approach our bifunctional peptides can offer—working synergistically with the SDF to help protect and rebuild damaged dental tissues while adding a new mineral layer that can be incorporated in adhesive dental composites to restore function and esthetics to children from underserved communities suffering from the epidemic of early childhood caries.

Previously, our group has demonstrated bifunctional capabilities of our solid binding peptides in the oral cavity acting to combat oral bacteria, direct remineralization of dentin and enamel, and as bioactive components of engineered dental biomaterials to improve binding and native tissue integration [44,45,46,47,50,51,52]. Limited studies have been published to date focused on masking or reducing the staining of SDF on carious dentin and enamel; they have thus far involved using potassium iodide, composites, or glass ionomer cements to address this [84,85,86]. Hamdy et al. used a calibrated spectrophotometer to monitor changes in tooth color following SDF treatment and found a composite coating was the most successful masking agent at baseline and after an aging protocol of the three treatments [85]. However, the composite treatment that immediately followed SDF application required an etching step and multiple coats of dental composite on the affected surface and the effectiveness of the initial SDF treatment after the composite coverage was not reported. Furthermore, it is known that composite restorations in the oral cavity have a high rate of failure due to cracks and gaps creating spaces for bacterial infiltration causing further decay [87,88,89,90]. With the selective and targeted anchoring peptide, AgBP2, one potential improvement to previous studies would be to include our peptide within the adhesive to help reinforce interfacial binding between the SDF-treated dental surface and composite restoration. Continuing our recent exploration of the vast potential of bioactive peptides to improve interfacial integrity and binding of polymeric composites to dental surfaces, we show the targeting capability of AgBP2 for SDF-treated regions in carious dental tissues [47,50,51,52,91].

Another approach to mask or reduce the staining caused by SDF has focused on remineralization of the affected dentin and enamel. Bacteria associated with dental caries works to break down the tooth structure, and the fluoride component of SDF treatment promotes natural remineralization of a treated tooth [25,82,92]. We, and colleagues, have previously established multiple peptides capable of directing remineralization of calcium phosphate materials and thus wanted to explore a synergistic approach using bifunctional peptides to anchor to the SDF-treated regions and direct new mineral growth [44,45,51,52,61,77]. The iterative spacer design process of novel, bifunctional peptides featuring AgBP2-anchoring peptide for targeted binding to SDF is also being studied to ensure optimal and tunable activity of both active domains.

## 5. Conclusions

Fluorides have proven useful to slow the progression of dental caries by inhibiting the demineralization of teeth. Recently, silver diamine fluoride (SDF) has received attention as a non-invasive, inexpensive, and easy to use approach for managing cavitated lesions in the teeth of children. SDF was approved for dental use by the FDA in 2014, however, widespread adoption of SDF has been slow because SDF-treated caries affected dental tissue are stained black. Herein, we report for the first time the specific binding of a silver-binding peptide to silver diamine fluoride (SDF)-treated human dental tissues. Building upon the success of anchoring peptide onto silver stain deposits, we next computationally engineered a bifunctional peptide composed of silver-binding peptide with an amelogenin-derived peptide (shADP) domain separated by a spacer sequence. Amelogenin protein, recognized for its role in guiding enamel formation and mineralization, was mimicked by the incorporation of shADP peptide to induce fast mineralization on the dental tissues. Computational folding investigation of the bifunctional peptides with different spacers were utilized to preserve the function of both peptide domains. The designed novel shADP5-AgBP2 bifunctional peptide was demonstrated to possess the ability to bind to SDF-treated dental tissues and to guide the formation of calcium phosphate nanocomposite materials on the dental tissue–peptide interface. Our results offer a peptide-enabled interface nanocomposite design to target the black staining associated with SDF treatment. Future studies must be conducted to tune the peptide sequence to achieve desired mineral composition and morphology to work synergistically with SDF to remineralize affected dentin and enamel. Additional work will be focused on employing novel dental adhesives with active AgBP-binding domains as an alternative approach to mask SDF staining. This work demonstrates bifunctional peptides as promising molecular agents to specifically bind to SDF-treated dental tissues as a novel modular and easily deployable approach to arrest the adverse impact of rampant childhood caries progression while preventing black staining due to SDF treatment.

## Figures and Tables

**Table 1:** Distances between AgBP2 and shADP5 compared with the bifunctional peptides by the mean sum of squares across the single-domain residues. Summed backbone change (%) is the combined change including both domains.

Name	Sequence	Summed Backbone Change (%)
shADP5	SYEKSHSQAINTDRT	N/A
AgBP2	EQLGVRKELRGV	N/A
shADP5_EAAAK_AgBP2	SYEKSHSQAINTDRTEAAAKEQLGVRKELRGV	9.4
shADP5_AgBP2 (No Spacer)	SYEKSHSQAINTDRTEQLGVRKELRGV	14
shADP5_APA_AgBP2	SYEKSHSQAINTDRTAPAEQLGVRKELRGV	17
shADP5_GGG_AgBP2	SYEKSHSQAINTDRTGGGEQLGVRKELRGV	18
shADP5_PAPAP_AgBP2	SYEKSHSQAINTDRTPAPAPEQLGVRKELRGV	23
shADP5_GSGGG_AgBP2	SYEKSHSQAINTDRTGSGGGEQLGVRKELRGV	25

**Table 2:** Averaged calculated Ca/P ratios of dental tissues in untreated and SDF-treated regions in dentin and enamel across.

			Biomimetic Mineralization	
	Untreated Control	SDF-Treated Control	SDF-Treated with No Peptide	SDF-Treated with Bifunctional Peptide
Enamel	1.375	1.447	1.092	1.338
Dentin	1.405	1.419	1.066	1.291
SDF-treated enamel	n/a	1.540	1.100	1.257
SDF-treated dentin	n/a	1.546	1.137	1.238

## Data Availability

Not applicable.

## Supplementary Materials

The following supporting information can be downloaded at: https://www.mdpi.com/article/10.3390/polym14071368/s1, Figure S1: ATR-FTIR Spectrum Interaction of Silver Diamine Fluoride and Silver-Binding Peptide
