# Supplementary material for: Peptide-Enabled Nanocomposites Offer Biomimetic Reconstruction of Silver Diamine Fluoride-Treated Dental Tissues
Source: Polymers (Basel). 2022 Mar 28;14(7):1368. doi: 10.3390/polym14071368 (PMC9002525; doi:10.3390/polym14071368)
Supplement: Supplementary file 1 [file polymers-14-01368-s001.zip › polymers-1585625-supplementary.pdf]

Peptide enabled nanocomposites offer biomimetic reconstruction of silver diamine fluoride treated dental tissues

Sarah K. Woolfolk [1,2], Aya K. Cloyd [1,2], Qiang Ye [2], Kyle Boone[2,3]

Paulette Spencer [1,2,3], Malcolm L. Snead [1,4], Candan Tamerler [1,2,3]*

[1] University of Kansas, Bioengineering Program, 1530 W. 15th Street, Lawrence, KS 66045-7609, USA

[2] University of Kansas, Institute for Bioengineering Research, 1530 W. 15th Street, Lawrence, KS 66045-7609, USA

[3] University of Kansas, Department of Mechanical Engineering, 1530 W. 15th Street, Lawrence, KS 66045-7609, USA

[4] Center for Craniofacial Molecular Biology, Herman Ostrow School of Dentistry of USC, The University of Southern California, 2250 Alcazar Street, Los Angeles 90033-9062, USA

*** Corresponding author**

Candan Tamerler, PhD

Charles Y. and Mary J. Spahr Professor

Dept. Mechanical Engineering,

Institute for Bioengineering Research

The University of Kansas

1530 W. 15th Street, Lawrence, KS 66045-7609

Ph: 785-864-2984

Email: ctamerler@ku.edu

Supplemental Information:

Silver-Binding Peptide interacting with Silver Diamine Fluoride

We explored the interaction between silver diamine fluoride (SDF) and silver-binding peptide (AgBP) using attenuated total reflectance Fourier transform infrared spectroscopy (ATR-FTIR). The shifts in the frequency of absorption bands indicate presence of functional groups or changes on the surface chemistry on the sample. We investigated the interaction of SDF and AgBP directly.

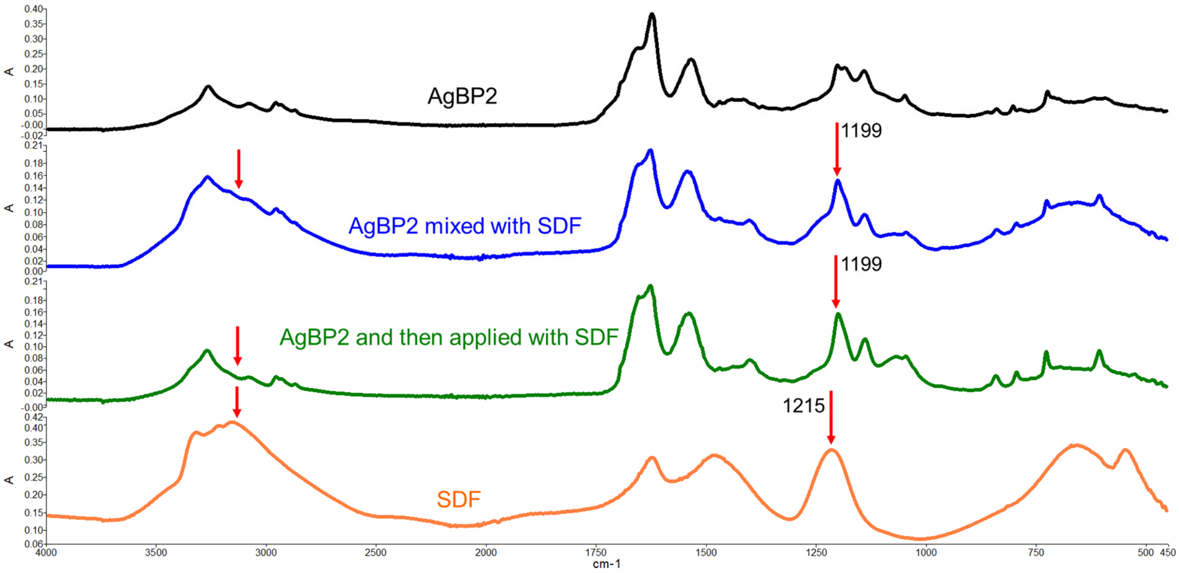

Figure S1. ATR-FTIR spectra of silver binding peptide (AgBP2) with and without interaction of silver diamine fluoride (SDF). With the presence of SDF, the spectra of AgBP2 shows increased intensity of N-H stretching vibration at 3100 cm-1. The significant peak shifting of N-H bending vibration from 1215 cm-1 to 1199 cm-1 indicated the interaction of AgBP2 and SDF.

Figure S1 provides the interaction of 4 mg/mL SDF and 4 mg/mL AgBP (stoichiometry 13 SDF complexes: 1 peptide). The SDF complex contains ammonia-based compounds, expected FTIR features include: N-H stretching 3600-3300 cm-1, H-N-H scissoring 1800-1600 cm-1, and N-H

wagging 1100-1300 cm-1. We see a substantial peak shift in the N-H bending vibration taking place in the presence of AgBP. This happens both with mixing the two components in solution and with applying AgBP first to the ATR crystal, then SDF. This peak shift demonstrates the interaction of AgBP and SDF.
